# Natural Product Synthesis Enabled by Radical-Polar Crossover Reactions

**DOI:** 10.1021/acs.joc.5c00306

**Published:** 2025-04-04

**Authors:** Nicolas Müller, Thomas Magauer, Ondřej Kováč

**Affiliations:** † Department of Organic Chemistry and Center for Molecular Biosciences, University of Innsbruck, 6020 Innsbruck, Austria; ‡ Department of Organic Chemistry, Palacký University Olomouc, 77900 Olomouc, Czech Republic

## Abstract

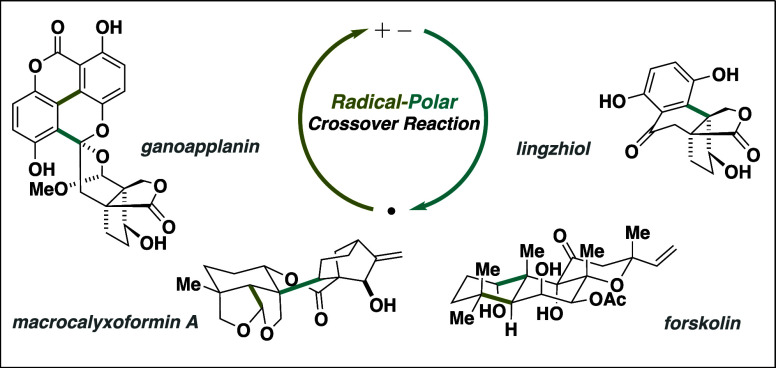

Radical-polar crossover (RPC) chemistry is an emerging field characterized by transformations that involve the coexistence of both radical and ionic species. Since the reactivities of radical and ionic intermediates are orthogonal, applying these two mechanisms in sequence provides significant advantages in the construction of complex molecular architectures. The concept of the RPC approach has become increasingly important in the total synthesis of natural products. This Synopsis presents several examples to showcase recent advancements in this area, including our research on the synthesis of *Ganoderma* meroterpenoids. In these selected cases, RPC reactions enhance the building of structural complexity and improve overall synthetic efficiency that cannot be achieved by standard synthetic methods.

Chemists have gained a solid understanding of polar reactions, which involve the transfer of electrons between or within molecules due to differences in electronegativity. However, radical reactions, characterized by the involvement of unpaired electrons, tend to be less studied within the chemical community. In recent years, a groundbreaking concept has gained significant attention: combining polar and radical intermediates into what are known as radical-polar crossover (RPC) reactions (Figure 1).

**Figure 1:**
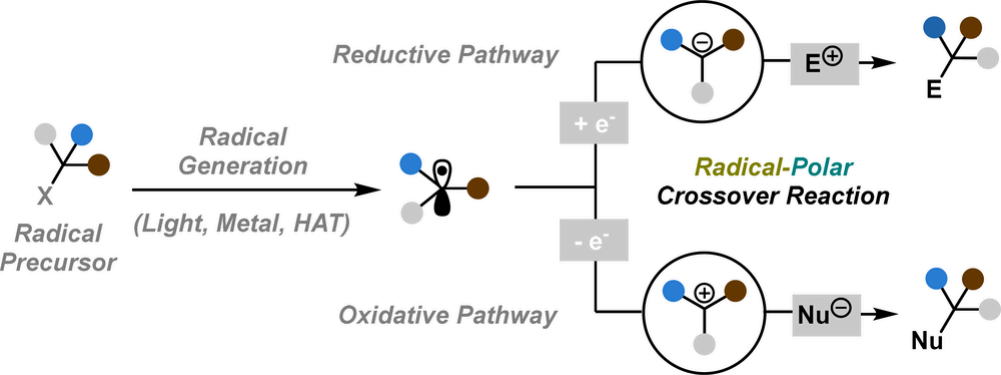
Radical-polar crossover (RPC) logic.

This concept is an invaluable tool for chemists, effectively bridging the gap between the traditional mechanisms of polar chemistry and the unconventional pathways associated with radical chemistry.^1−4^ By doing so, these approaches not only overcome the inherent limitations of each reaction type but also provide new avenues for other synthetic transformations. Additionally, the synthesis of complex molecules often requires more subtle reaction pathways, and these methodologies allow for such advancements by utilizing the unique characteristics of both radical and polar chemistry.^5^ In the past few years, there has been considerable development in this area, leading to the emergence of a variety of synthetically important transformations.^6^ This Account aims to summarize the key advancements in the area of natural product synthesis and highlight contributions made in this field.

This Synopsis is organized according to the following reaction types: 1) Boron-, Tin-, or Nickel-mediated RPC reactions; 2) Cobalt- or Iron-mediated RPC reactions; and 3) Light-mediated RPC reactions.

## Boron-, Tin-, or Nickel-Mediated Radical-Polar Crossover Reactions

Modern natural product synthesis should aim to minimize nonconstructive synthetic operations while maximizing generation of skeletal complexity in each step. One practical approach for this is multicomponent reactions, where multiple C–C or C–O bond-forming events can shorten the synthetic sequence and lead to an increased overall efficiency. Notably, this strategy could be combined within the realm of the RPC reactions, where the orthogonal coexistence of the radical and polar intermediates offers a powerful combination for forging multiple bonds in a one-pot manner.

**Scheme 1:**
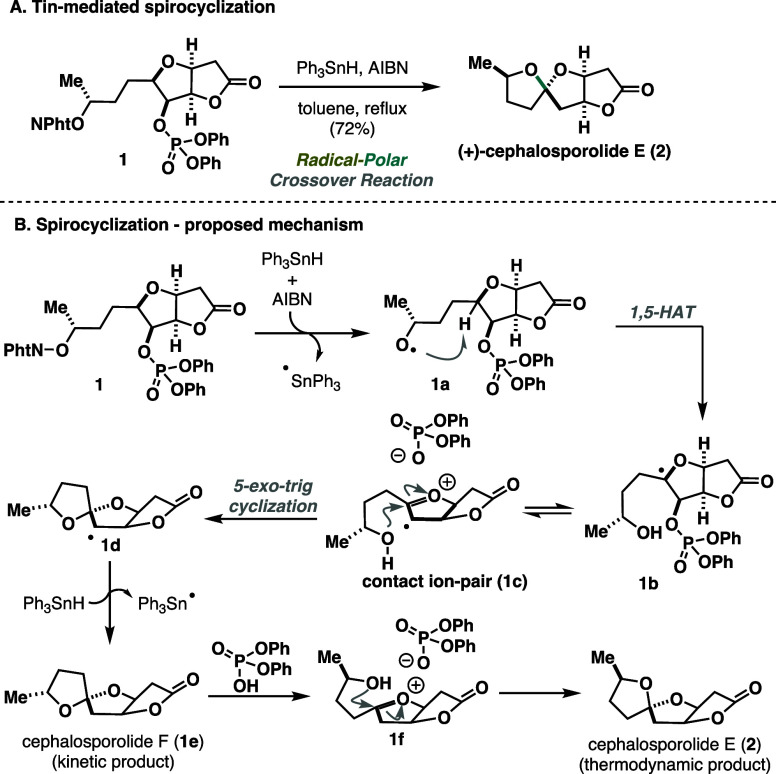
Sartillo-Piscil Synthesis of (+)-Cephalosporolide E (2015)

Natural products containing a 5,5-spiroketal moiety have received special attention due to their biological importance and structural complexity.^7^ Notably, the unique spiro motif poses a significant synthetic challenge due to the lack of stereocontrolled synthetic protocols.^8^ In 2015, Sartillo-Piscil introduced an innovative tandem sequence that leverages the RPC reaction to achieve the stereoselectivity of the 5,5-spiroketal moiety.^9^ This synthetic methodology proved highly effective, particularly in the synthesis of cephalosporolide E (**2**) showcasing its practical application in complex organic synthesis (Scheme 1A). Treatment of **1** with triphenyltin hydride (Ph_3_SnH) and the radical initiator 2,2’-azodi(isobutyronitrile) (AIBN) facilitated the key stereoselective spiro-cyclization and delivered the tricycle **2** in 72% yield. Mechanistically, the authors proposed an RPC pathway (Scheme 1B). Thus, the generated triphenyltin radical induces the formation of *O*-centered radical **1a**, responsible for 1,5-hydrogen atom transfer (HAT) that delivers **1b**. The equilibrium between **1b** and **1c** reveals an oxonium ion and a pendant alcohol group that readily cyclizes and gives rise to 5,5-spiroketal scaffold **1d**. The observed stereoselectivity could be explained by effective shielding of the top face of oxonium ion **1c** by the phosphate anion, forcing the hydroxyl group to react from the opposite side. The final stage includes hydrogen abstraction from Ph_3_SnH followed by diphenyl-phosphate acid (generated in situ) induced isomerization to thermodynamic product **2**.

**Scheme 2:**
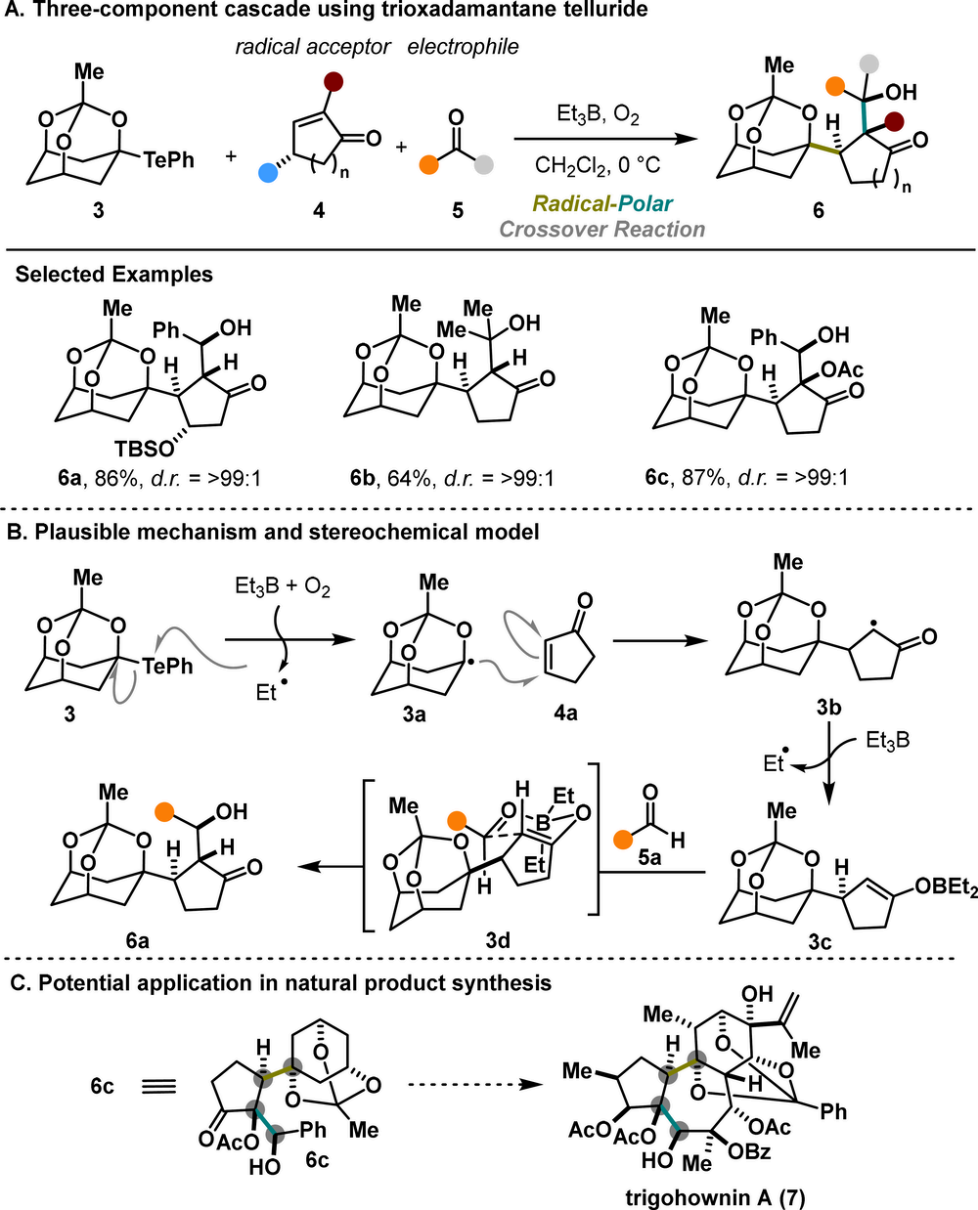
Inoue Three-Component Cascade (2013)

The Inoue group has developed several inspiring multicomponent coupling reactions and has shown their potential for natural product synthesis. In 2013, they reported the use of O–Te-acetal **3** as a radical precursor in a three-component cascade reaction including a Giese addition followed by aldol addition.^10^ This method tolerates various unsaturated ketones **4** as radical acceptors and carbonyl compounds **5** for the aldol step. Notably, the cascade efficiently installs three continuous stereocenters (Scheme 2A) with good to excellent control over the relative stereoconfiguration. The reaction pathway is presumably initiated via homolytic cleavage of the labile C–Te bonds upon treatment with BEt_3_/O_2_. The stable nucleophilic α-alkoxy bridgehead radical **3a** readily undergoes Giese addition with **4a**, generating a new radical species, **3b**. Moreover, the radical trapping capability of BEt_3_ is utilized in generating boron enolate **3c**, which then undergoes an aldol reaction to produce the desired product, **6a**. Notably, the six-membered transition-state model **3d** illustrated in Scheme 2B explains the impressive selectivity observed in this reaction. Specifically, a *trans* relationship between the radical acceptor and the electrophile is established by approaching from the opposite face of the bulky triox-adamantane structure. Overall, this cascade reaction can significantly enhance the molecular complexity in a single step, providing a valuable strategy for synthesizing functionalized terpenoids with intricate architectures (e.g., **7**, Scheme 2C). Despite the overall efficiency, the method does not offer variability of the radical precursors and remains limited to compound **3**.

**Scheme 3:**
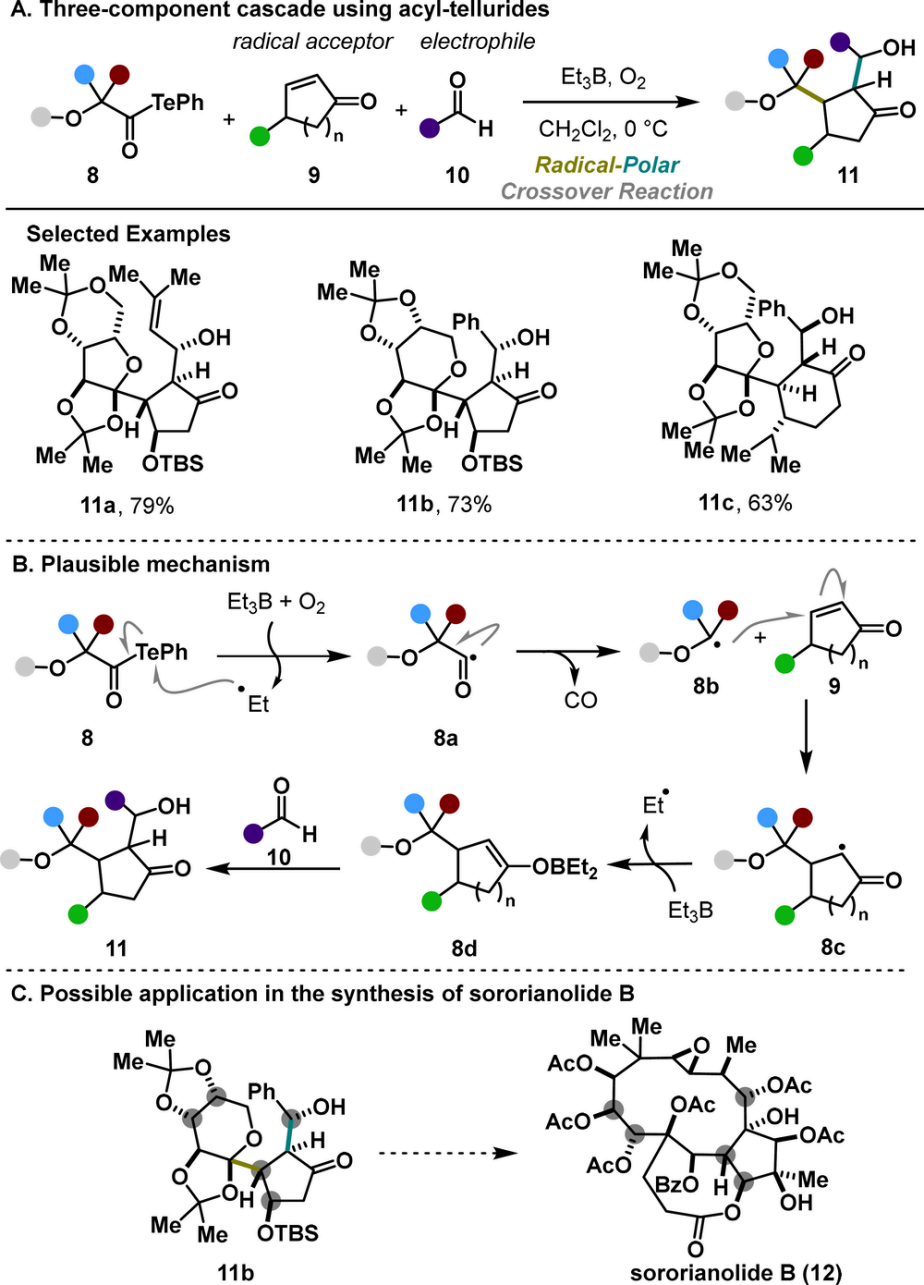
Inoue Three-Component Cascade with α-Alkoxyacyl Tellurides (2015)

The second installment from the same laboratory arose from slightly modifying the telluride intermediate.^11^ Adding a carbonyl moiety to its structure resulted in a higher stability of starting acyl tellurides **8**, expanding the substrate scope (Scheme 3A). Remarkably, various oxidation patterns are well-tolerated, enabling the rapid assembly of oxygen-dense, highly functionalized scaffolds with complete control over the relative stereochemistry of four stereocenters. This methodology leverages the facile radical decarbonylation of α-alkoxy acyl tellurides under BEt_3_/O_2_ treatment. From a mechanistic point of view, the transformation is initiated with the generation of the acyl radical **8a** that collapses, upon the release of carbon monoxide, to the alkoxy radical **8b**. A subsequent Giese addition, followed by an aldol reaction, yielded the final product **11** (Scheme 3B). Potentially, the developed protocol positions itself as an advantageous approach toward various natural polyols, such as sororianolide B (**12**, Scheme 3C). Conversely, the substrate scope is limited to the stabilized α-alkoxy radicals, nontrivial radical precursors, and simple carbonyl compounds used in the aldol step.

**Scheme 4:**
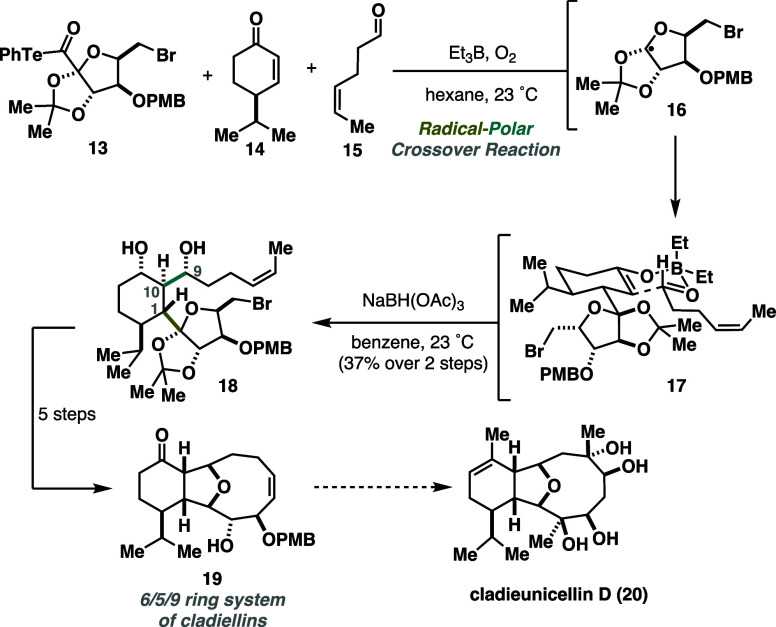
Inoue Three-Component Cascade Towards the Cladiellins Ring System (2019)

Additionally, Inoue’s three-component cascade proved to be effective for constructing the highly oxygenated 6/5/9 ring system of cladiellins **19** (Scheme 4). The previously employed mixture of BEt_3_ and O_2_ allowed efficient assembly of three fragments: **13** (radical precursor), **14** (radical acceptor), and **15** (electrophile). High stereoselectivity arose through rigid six-membered transition states, forging the consecutive stereocenters at positions C1, C9, and 10. Notably, the presence of bromide in the molecule was tolerated, and halogen remained intact.^12^

The key three-component cascade was immediately followed by ketone reduction to prevent an undesired retro-aldol side reaction. Overall, the complex 6/5/9 polycyclic structure was rapidly accessed in 12 steps from commercially available starting materials, serving as a potentially valuable late-stage intermediate for the synthesis of cladieunicellin D (**20**). The RPC reaction played a key role in the rapid construction of a densely functionalized structure, which would be challenging to obtain using conventional methods (either polar or radical reactions). Further applications in the target-oriented synthesis can be expected.^13,14^

**Scheme 5:**
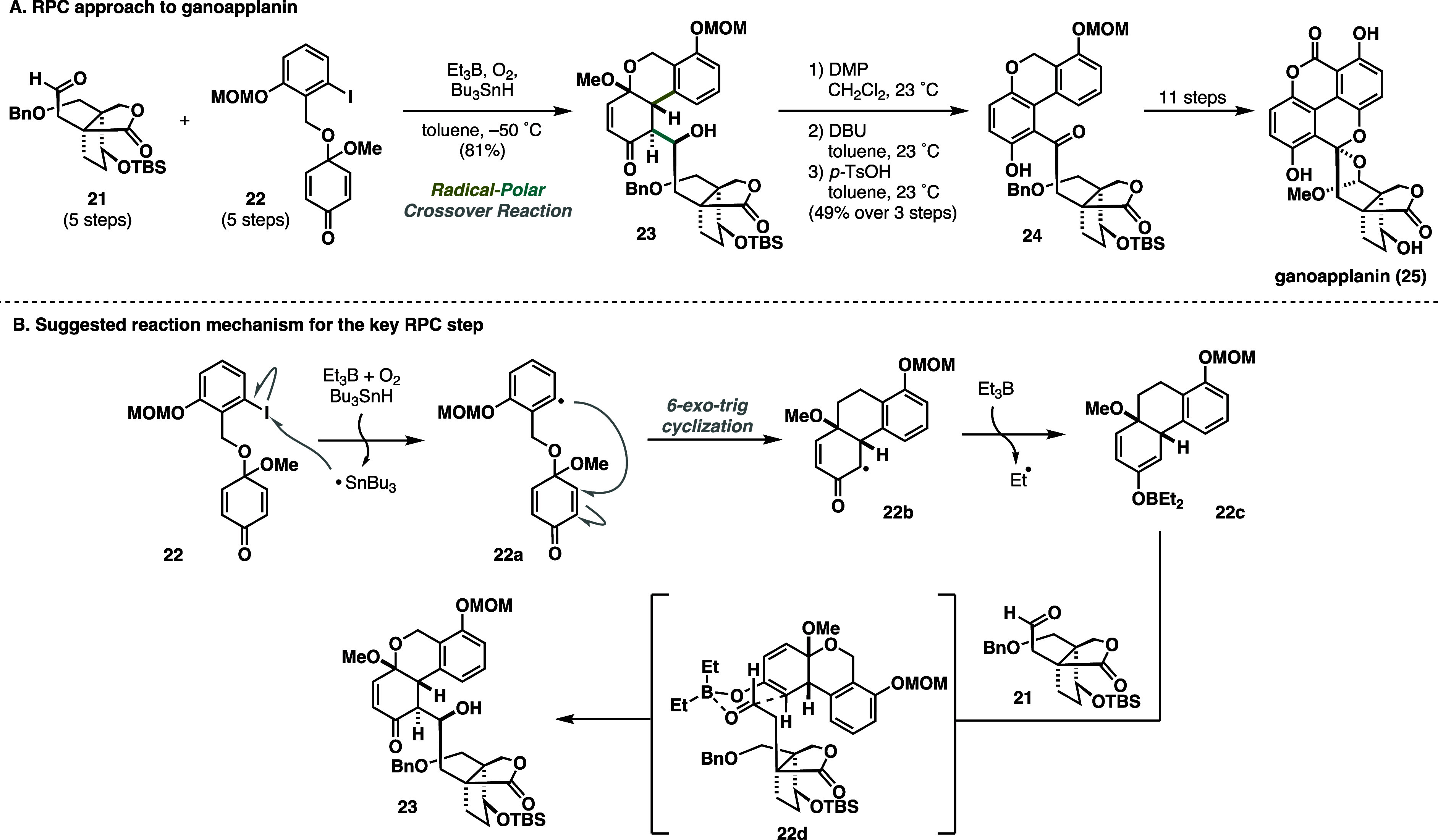
Magauer Synthesis of Ganoapplanin (2024)

The Magauer laboratory has made another innovative contribution to the RPC field, showcasing a convergent and efficient total synthesis of ganoapplanin (**25**) (Scheme 5A).^15,16^ The unique structural features of ganoapplanin (**25**) set it apart from other meroterpenoids, characterized by an unprecedented spiro bisacetal motif embedded within a 6/6/6/6 tetracyclic system, a tetra-*ortho* substituted biaryl motif, and a dioxatricyclo[4.3.3.0] dodecane scaffold.^17^ In this example, a boron- and tin-mediated RPC cascade to construct the complete carbon skeleton rapidly in a single step was devised. Gratifyingly, a fusion of aldehyde **21** and aromatic fragment **22** was enabled under modified conditions reported by Inoue via Giese-type cyclization immediately followed by an intermolecular aldol step.^11^ Subsequently, the oxidation pattern adjustment yielded the late-stage intermediate **24** that was advanced toward ganoapplanin (**25**) in 11 steps (Scheme 5A). The mechanism of the key transformation presumably followed a generally accepted pathway in which Giese cyclization is initiated via halogen abstraction, inducing 6-*exo*-trig cyclization. Radical **22b** is trapped by BEt_3_, generating the boron enolate **22c**, which binds to aldehyde **21** in a six-membered transition state **22d**, facilitating the aldol step (Scheme 5B). This cascade’s unique utility demonstrates the RPC reaction’s power in C–C bond-forming events that are otherwise challenging to achieve under typical polar, transition-metal, or radical reaction conditions.

**Scheme 6:**
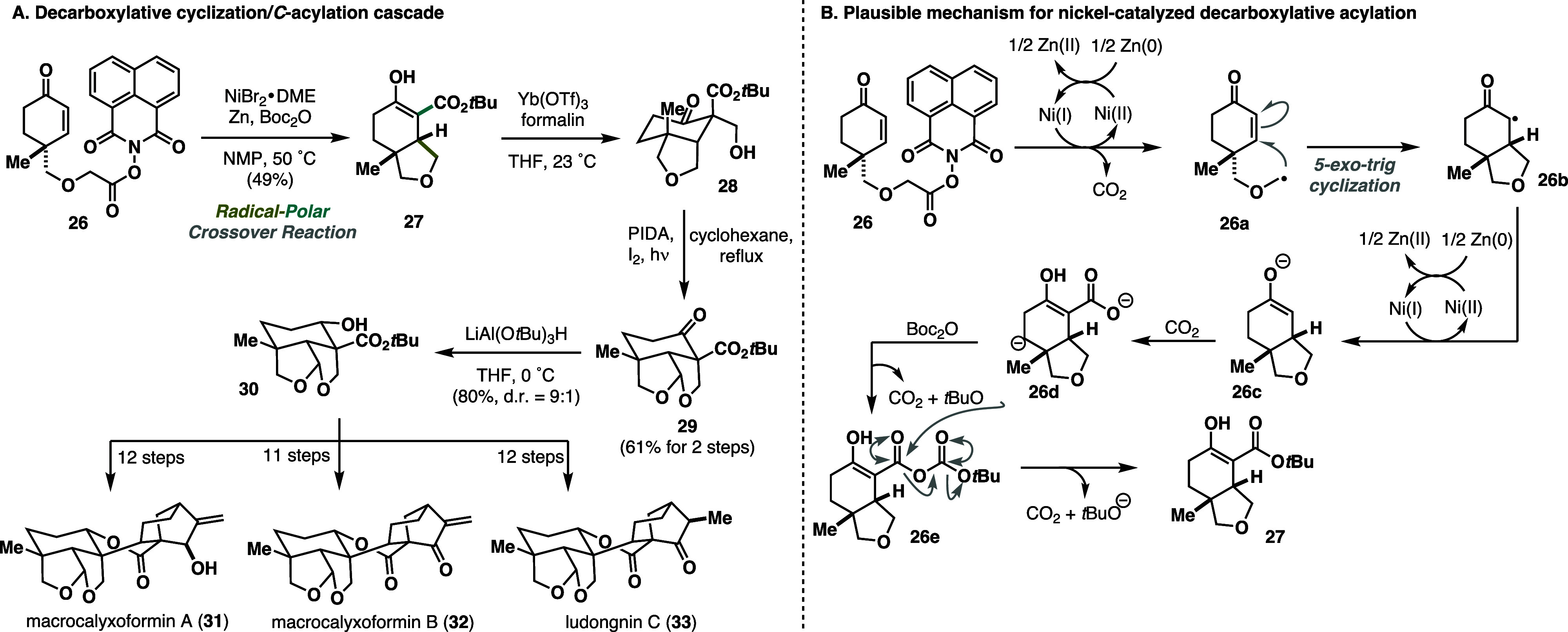
Li Collective Synthesis of Macrocalyxoformin A-B and Ludongnin C (2024)

Recently, Li published an elegant synthesis of several *ent*-kauranoids natural products, including macrocalyxoformin A–B (**31**, **32**) and ludongnin C (**33**).^18^ One of the highlights of the synthesis was the use of a nickel-catalyzed decarboxylative cyclization/radical-polar crossover/*C*-acylation cascade to forge fused THF ring **27**. Despite its overall efficiency, this strategy presented considerable challenges that needed to be addressed, particularly the choice of the acylation reagent (*C* vs *O*-acylation) and the chemoselectivity of the acylation. Extensive optimization using various redox-active esters, nickel sources, ligands, temperature, and acylation agents revealed substrate **26** as optimal for the desired decarboxylative cyclization-acylation sequence. Thus, treatment with a nickel(II)bromide 1,2-dimethoxyethane complex, zinc, and Boc-anhydride (Boc_2_O) in *N*-methyl-2-pyrrolidine (NMP) at 50 °C afforded the desired bicyclic THF scaffold **27** in 49% yield with excellent stereoselectivity (Scheme 6A). To gain insight into this transformation, the authors performed experiments with ^13^C-labeled substrates and reagents, leading to surprising findings. The initial steps involve the reduction of nickel(II) to nickel(I), facilitating reductive decarboxylation with the concomitant formation of radical **26a**. Subsequent Giese 5-*exo*-trig cyclization led to the bicyclic motif **26b**, which was readily reduced to the enolate in the presence of nickel(I). Surprisingly, findings revealed that carbon dioxide released at the initial phase of the sequence is presumably trapped by enolate **26c**, generating carboxylate **26d** that is then activated with Boc_2_O. The final step includes decomposition of the anhydride, releasing desired product **27** from the catalytic cycle (Scheme 6B). A further sequence of aldol reaction, Suaréz oxidation, and reduction yielded tricycle **30**, which was subsequently elaborated into the final natural products in 11 (**32**) or 12 (**31**, **33**) steps, respectively.

## Cobalt- or Iron-Mediated Radical-Polar Crossover Reactions

Hydrogen atom transfer (HAT) reactions have emerged as a powerful method for the selective C–H, C–O, and C–C bond formation.^19,20^ Its unique chemoselectivity and unprecedented retrosynthetic possibilities have allowed concise approaches to several complex natural products.^21−24^

**Scheme 7:**
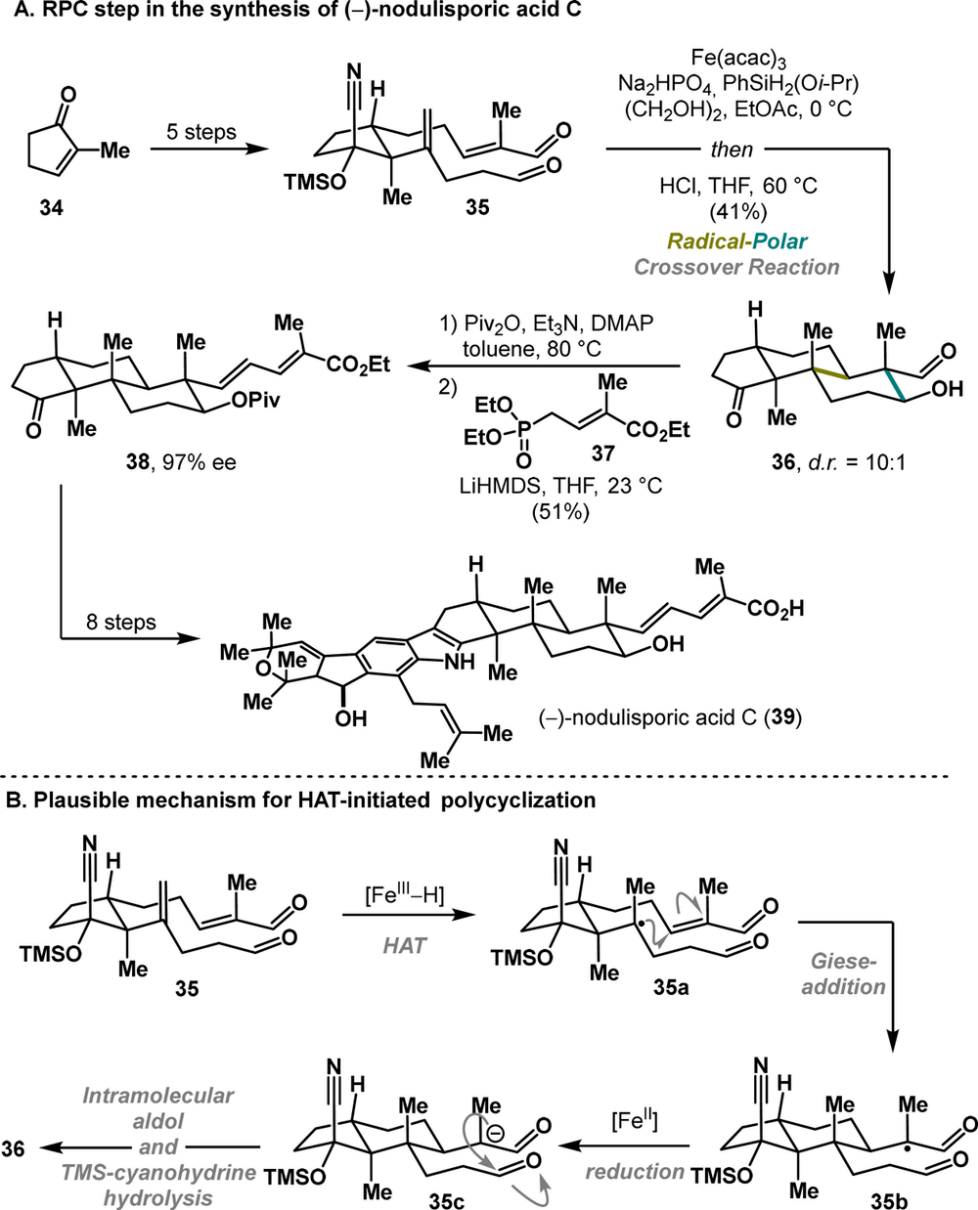
Pronin Synthesis of (−)-Nodulisporic Acid C (2018)

In 2018, Pronin and co-workers demonstrated the utility of HAT-initiated RPC in novel polycyclization reactions, enabling the synthesis of (−)-nodulisporic Acid C (**39**) in just 12 steps.^25^ The synthesis commenced from a simple unsaturated ketone **34** that was elaborated into the cyclized precursor **35** in 5 steps. Subjecting **35** to iron-mediated HAT^26,27^ facilitated a diastereoselective cyclization (d.r = 10:1) to the *trans*-decalin fragment **36** (Scheme 7A). Interestingly, the presence of the TMS-cyanohydrin moiety proved crucial, as omission of the pseudoaxial substituent (CN) led to significant erosion of the diastereoselectivity.^21^ Mechanistically, the reaction is initiated via an iron-mediated hydrogen transfer to the double bond, forming the stable tertiary radical **35a**. The close proximity of the electron-deficient double bond, enabled by a chairlike conformation, facilitates intramolecular Giese cyclization. Subsequent reduction, followed by an intramolecular aldol reaction, completes the annulation cascade.^21^ The final acidic workup revealed tricyclic intermediate **36**. The synthesis of (−)-nodulisporic acid C (**39**) was completed in just eight steps from **38**, demonstrating the remarkable efficiency of the key RPC annulation.

**Scheme 8:**
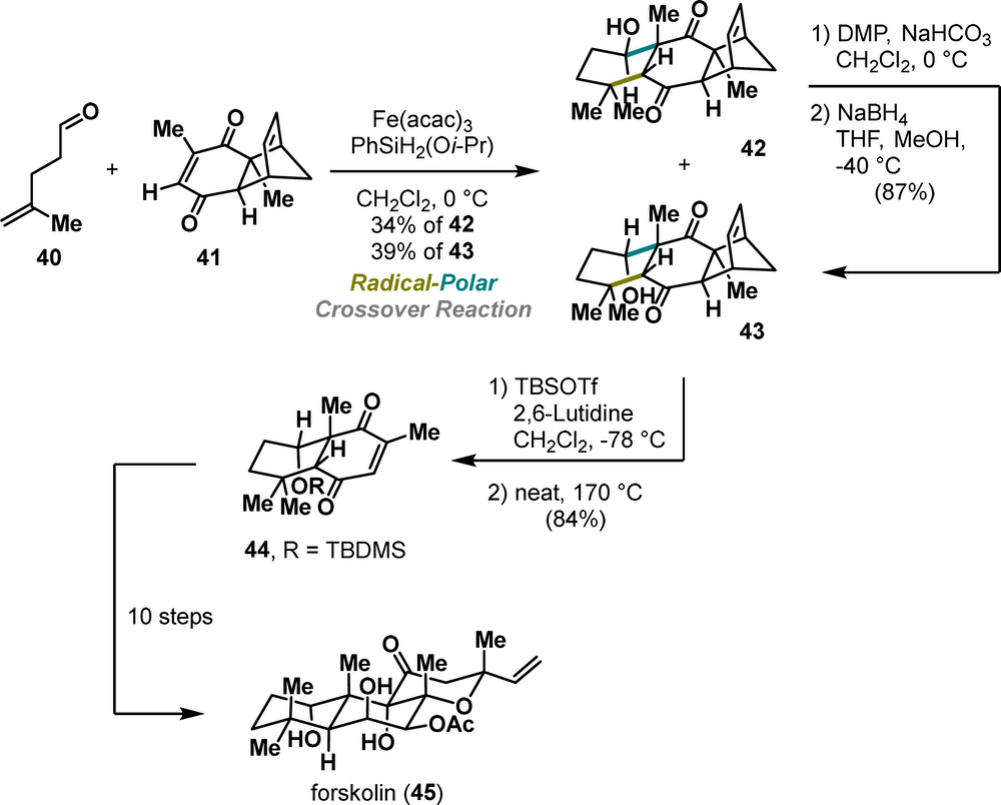
Pronin Synthesis of Forskolin (2019)

The same research group advanced the annulation cascade by enriching the protocol in favor of an intermolecular version. Its utility was demonstrated in an exceptionally short synthesis of the terpenoid natural product forskolin (**45**) (Scheme 8).^28^ Employing aliphatic aldehyde **40** and readily available enone **41** in the iron-mediated RPC annulation cascade smoothly provided desired polycyclic motifs **42** and **43** in an overall 73% yield.

Despite the diminished diastereoselectivity (dr = 1:1), the process proved to be robust and scalable. Conversely, the undesired diastereomer **42** could be further elaborated into the desired product **43** via an oxidation–reduction sequence, selectively yielding the preferred diastereomer.

Subsequently, protection of secondary alcohol followed by a thermally induced retro Diels–Alder reaction revealed quinone **44** that was further advanced to the forskolin (**45**) in 10 steps. Mechanistically, products **42** and **43** are formed via similar pathways (HAT–Giese addition–aldol reaction) depicted in Scheme 7B. Importantly, this RPC approach represents an attractive complementary approach for assembling six-membered carbocycles that are otherwise challenging to obtain via conventional methods (e.g., the Diels–Alder reaction), significantly expanding the synthetic toolbox.

**Scheme 9:**
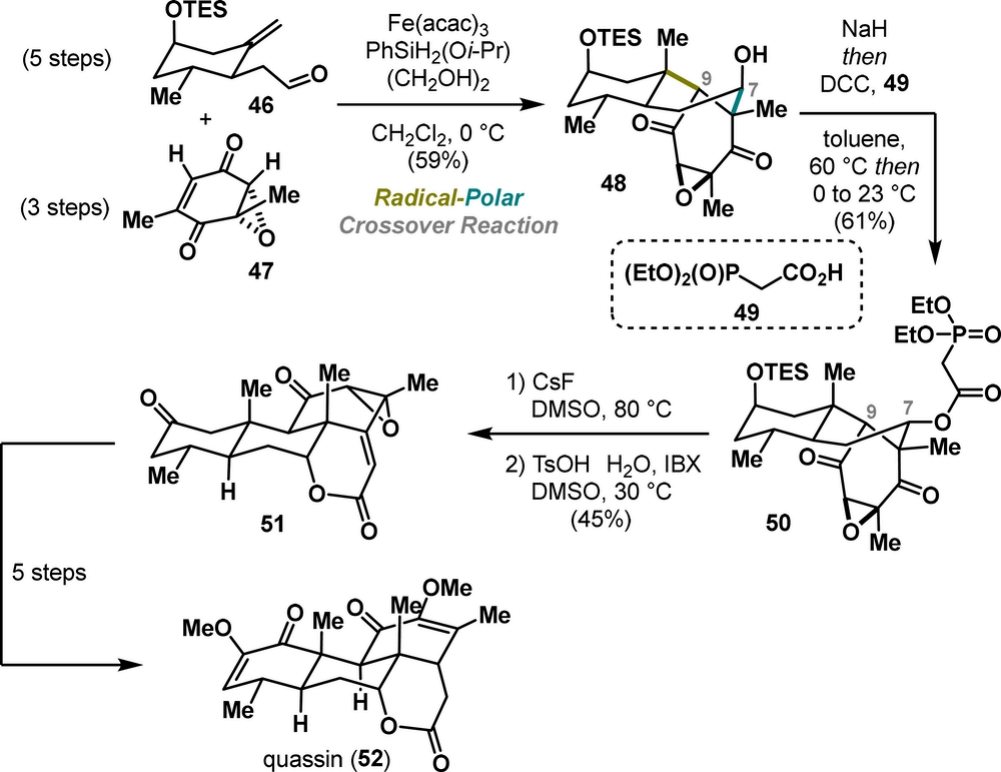
Pronin Synthesis of Quassin (2022)

A few years later, Pronin greatly simplified access to quassinoid natural products, terpenoids with diverse biological activities, such as potent cytotoxicity and antimalarial activity against drug-resistant strains.^29^ Driven by the limited supply of further biological studies, Pronin devised a concise approach to the polycyclic motif of quassinoids. The key step involved a carefully designed reaction between aldehyde **46** and epoxy-quinone **47**, yielding the desired product **48** in 59% yield (Scheme 9). Notably, comparing the epoxide’s reactivity with that of the previously used quinone **41** (Scheme 8),^28^ the epoxide **47** performed substantially better, concerning stereoselectivity during the aldol step.^30^ Despite the excellent diastereoselectivity, secondary alcohol **48** exhibited an opposite configuration at C7. Nevertheless, conformational analysis revealed that intermediate **48** adopted a twist-boat conformation; therefore, epimerization of the pseudoaxial alcohol could be achieved via a retro-aldol/aldol sequence. Indeed, the stereochemical outcome was successfully inverted using sodium hydride, followed by the addition of diethylphosphonoacetic acid that provided **50** with the correct configuration at C7. The successful installation of the phosphonate ester enabled the sequence to proceed with an intramolecular Horner–Wadsworth–Emmons reaction. Ultimately, treating **50** with cesium fluoride induced intramolecular olefination to deliver the complete polycyclic scaffolds of quassinoids **51** (Scheme 9). The endgame of the synthesis required five additional steps to obtain the target natural product quassin (**52**). Overall, the developed annulation strategy offers a valuable entry point to quassinoids, significantly reducing functional group manipulation. Moreover, this approach holds great potential for the synthesis of other congeners.

**Scheme 10:**
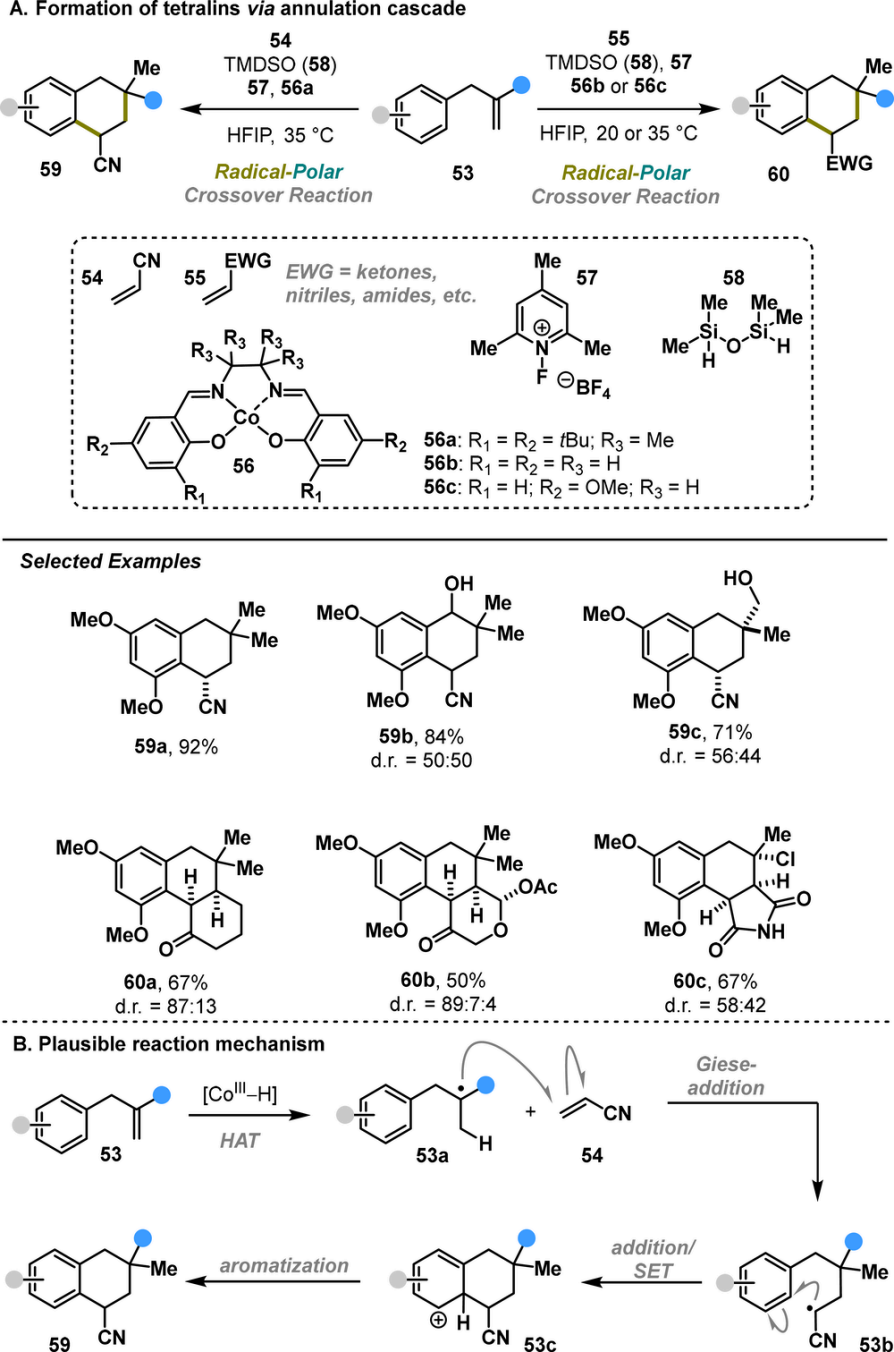
Vanderwal Annulation Cascade (2023)

Vanderwal reported the expansion of RPC methodology to the synthesis of complex tetralins.^31^ Despite the ubiquitous presence of complex tetralins in the structures of secondary metabolites and pharmaceutical compounds, direct strategies for constructing this motif have remained underdeveloped.^32,33^ Vanderwal developed a RPC protocol in which a cobalt-catalyzed hydrogen atom transfer facilitates tandem radical C–C bond formation, enabling the direct and concise synthesis of functionalized tetralins **59** and **60** (Scheme 10A). Evaluation of the scope and limitations of the method revealed a dependence on the nature of radical acceptors **54** or **55**, specific catalysts (**56a**–**c**), and different temperatures (20 or 35 °C). Nevertheless, a relatively broad substrate scope was achieved, with yields ranging from 26 to 92% and diastereoselectivity varying from poor to excellent. The proposed mechanism follows a pathway similar to that described above: 1) cobalt-mediated HAT, 2) Giese addition to Michael acceptor, 3) radical addition/oxidation, and 4) aromatization (Scheme 10B). The drawbacks of the method include poor diastereoselectivity and reliance on specialized reagents. Despite the limitations, this method offers an operationally simple synthetic protocol analogous to a formal [4 + 2] cycloaddition, which would be highly challenging to achieve under classical conditions.

## Light Mediated Radical-Polar Crossover Reaction

Photochemically induced RPC reactions represent a valuable alternative to the methodologies presented above. Recent advancements in photoredox catalysis have led to significant improvements, including milder reaction conditions, better functional-group tolerance, and reduced waste.^1,2^ Hence, exploiting such protocols could significantly improve the synthesis of complex natural products by minimizing functional group manipulations and eliminating nonconstructive steps.^34^

**Scheme 11:**
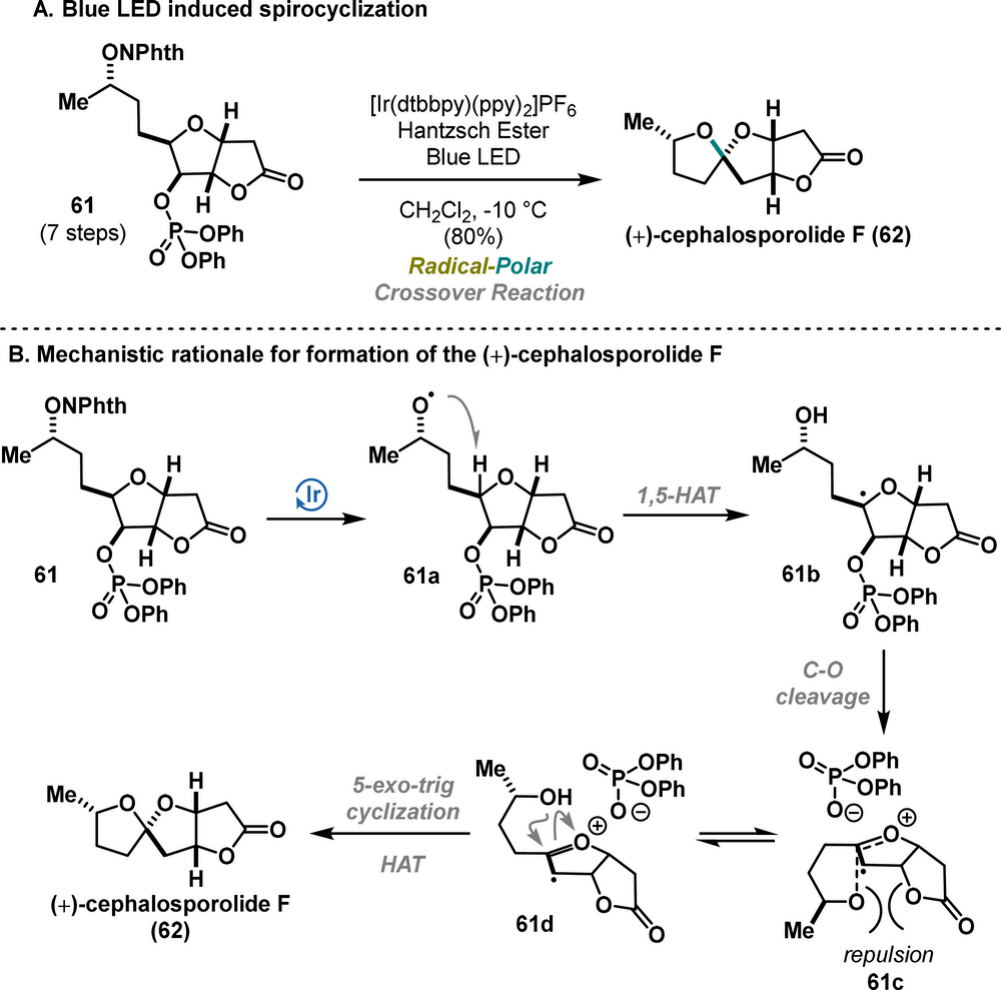
Sartillo-Piscil Synthesis of (+)-Cephalosporolide F (2023)

Recently, Sartillo-Piscil published an improved synthesis of (+)-cephalosporolide F (**62**), enabled by blue light-mediated RPC spiro cyclization as the final step of the synthesis (Scheme 11A).^35^

Thus, radical precursor **61** was treated with an iridium(III) catalyst in the presence of Hantzsch ester under blue LED light irradiation, facilitating the formation of the characteristic 5,5-spiroketal scaffold with excellent stereoselectivity and yield (80%).

A proposed stereoelectronic model depicted in **61c**/**61d** explains the preferred attack of the secondary alcohol. Under photochemical settings, an *O*-centered radical **61a** is formed, followed by the generation of the contact-ion pair **61c** via 1,5-HAT and C–O cleavage of the phosphonate. Destabilization of **61c** via Pauli repulsion provides radical cation **61d**, trapped by the hydroxyl group, enabling the stereoselective formation of (+)-cephalosporolide F (**62**) (Scheme 11B). In contrast to the findings of the 2015 report, compound **62** emerges as a challenging kinetic product, when compared to the thermodynamic product **2** presented in Scheme 1. This protocol, therefore, represents a significant advancement in the field as an alternative to the currently prevalent thermodynamically driven synthetic methodologies.^8^

**Scheme 12:**
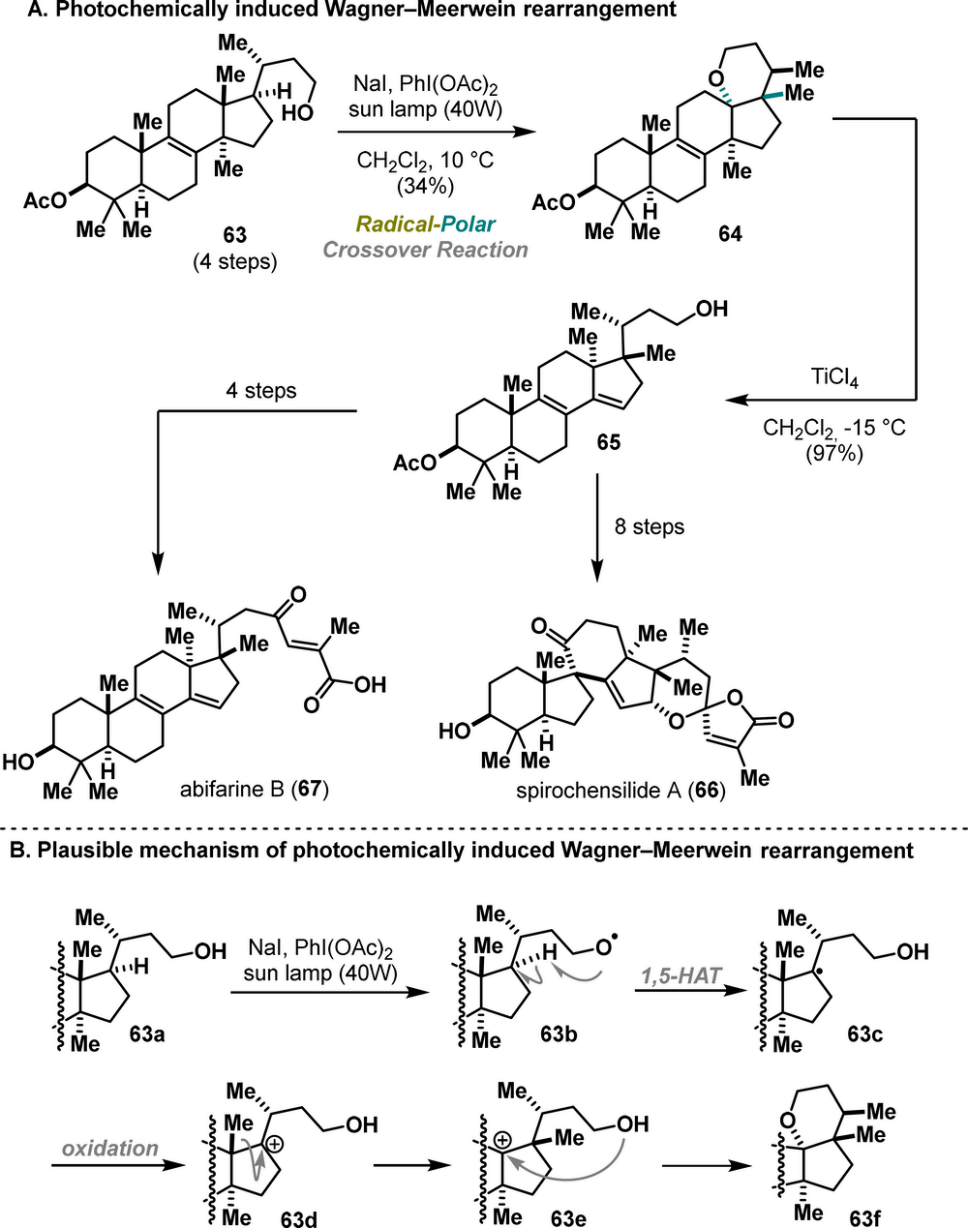
Heretsch Synthesis of Abifarine B and Spirochensilide B (2022)

In 2022, Heretsch provided an elegant example of radical-polar crossover processes under UV light.^36^ Inspired by a sequence of rearrangements proposed in the biosynthesis of spirochensilide A (**66**) and abifarine B (**67**), Heretsch and co-workers initiated a synthetic program to mimic these processes in the laboratory and to support the biogenetic hypothesis.^37^ The key precursor was prepared in 4 steps and subsequently rearranged into product **64** upon UV light irradiation by treatment with PIDA and NaI as an additive in 34% yield. Unfortunately, the methanide-shifted product could not be obtained in a higher yield, as various reaction conditions previously used to generate alkoxy radicals (e.g., HgO, Pb(OAc)_4_, PIFA with I_2_, or photocatalytic protocols after derivatization) proved to be insufficient. Nevertheless, substrate **64** was further elaborated using a second Wagner–Meerwein rearrangement, furnishing late-stage friedolanostane-type scaffold **65**. The desired natural products were successfully achieved in either four or eight steps (Scheme 12A). Mechanistically, the key transformation proceeds via an alkoxy radical intermediate, enabling 1,5-HAT. The resulting tertiary radical undergoes oxidation, triggering a methanide shift that leads to carbenium ion **63e**. The final intramolecular cyclization forms tetrahydropyran **63f** (Scheme 12B).

**Scheme 13:**
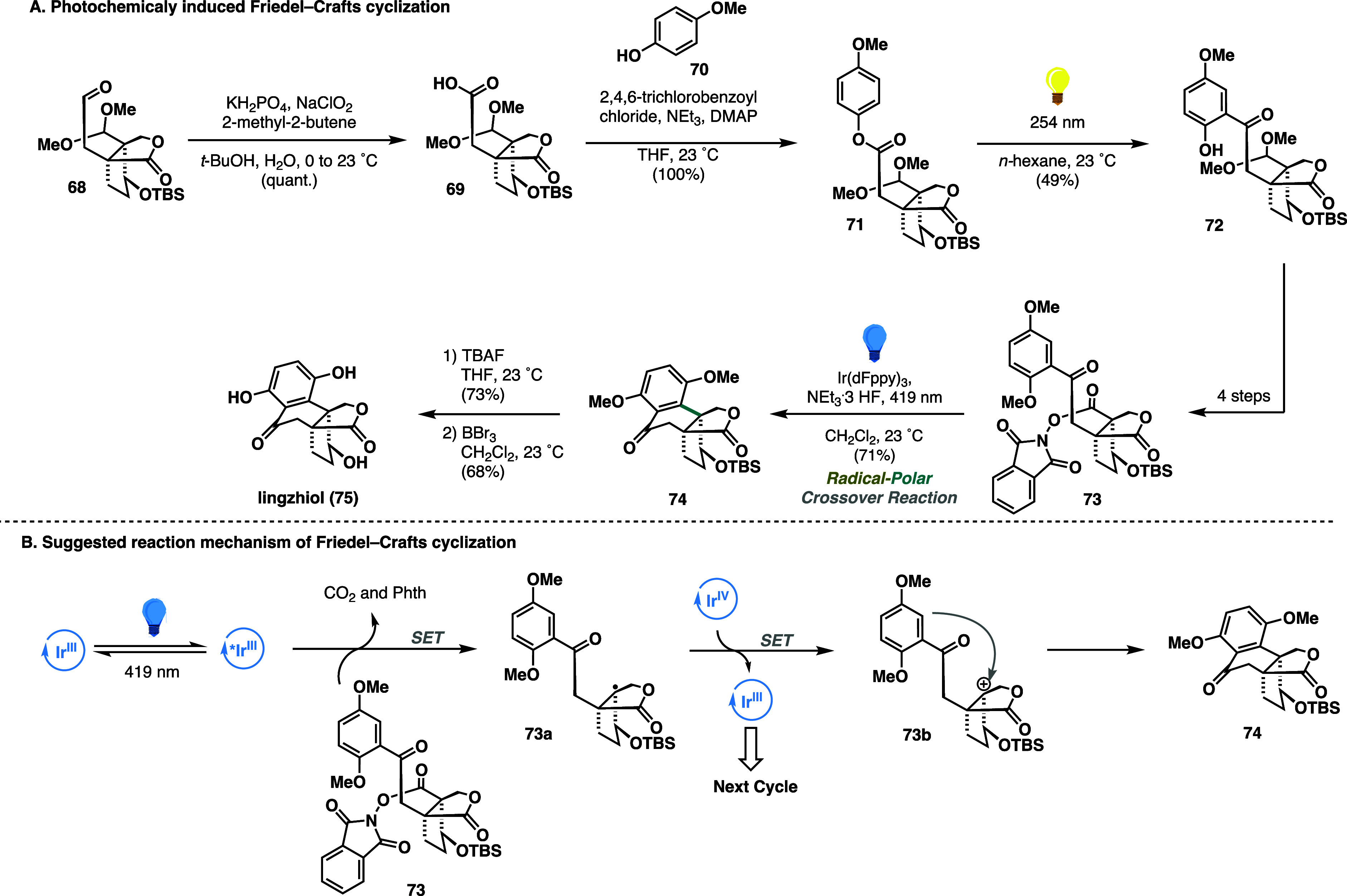
Magauer Synthesis of Lingzhiol (2024)

In 2024, Magauer contributed to this field by further expanding the repertoire of possible RPC strategies to access *Ganoderma* meroterpenoids. Overall, the efforts culminated in the development of a unified strategy enabling the concise synthesis of *six natural products* where synthesis of the most complex member linghziol (**75**) was enabled by a photoredox RPC reaction.^38^ With a sufficient quantity of bicyclic lactone **68** possessing the proper oxidation pattern, a Pinnick oxidation, followed by Yamaguchi esterification, allowed access to the key substrate **71** for the first photochemical transformation: a photo-Fries rearrangement.^39^ Thus, upon irradiation of compound **71** in *n*-hexane using a 254 nm wavelength at 23 °C, the formation of desired product **72** was observed. Encouraged by the efficiency of the rearrangement, **72** proceeded to the key RPC event (Friedel–Crafts cyclization). Based on the seminal work of Doyle on photocatalytic fluorination using redox-active esters,^40^ substrate **72** was first converted to the desired intermediate **73** in 4 steps. Gratifyingly, treatment of **73** with an Ir(dFppy)_3_ as a catalyst in combination with a catalytic amount of NEt_3_·3HF under 419 nm (blue light) irradiation led to facile cyclization, affording **74** in 71% yield. Finally, a deprotection sequence advanced the complete polycyclic structure to the natural product linghziol (**75**) (Scheme 13A). Based on previously proposed mechanistic rationales, a similar pathway involving single-electron reduction, followed by the extrusion of phthalimide and CO_2_, was suggested. The resulting tertiary radical **73a** undergoes oxidation via a second single-electron transfer, with subsequent cation trapping by an electron-rich aromatic ring yielding the tetralone motif **74** (Scheme 13B).

## Summary

We have highlighted the extraordinary potential of radical-polar crossover (RPC) reactions in the synthesis of natural products, particularly in the construction of their intricate molecular scaffolds. The integration of radical and polar intermediates in a one-pot manner can lead to fascinating mechanistic pathways, groundbreaking transformations, and improved synthetic efficiency. Notably, the implementation of mild photochemical or hydrogen atom transfer (HAT)-mediated radical-polar crossover sequences has emerged as a powerful strategy for organic chemists. While these methods have shown great promise in total synthesis, their full scope and potential remain largely untapped. Notably, the application of the RPC logic to alkaloid synthesis tends to be less developed, with great potential for future endeavors.^41,42^ Only by actively exploring and refining these techniques can we continue to advance the boundaries of RPC reactions in organic synthesis, unlocking potential applications in the creation of complex natural products.

## Data Availability

The data underlying this study are available in the published article.
